# Impacto de la calidad de vida relacionada a la salud en pacientes pediátricos con trasplante de progenitores hematopoyéticos en una institución colombiana

**DOI:** 10.7705/biomedica.6403

**Published:** 2022-09-02

**Authors:** Diego Medina-Valencia, Alejandro Castillo-Martínez, Estefanía Beltrán, Eliana Manzi, Amparo Chantre-Mostacilla, Gloria Piedad Guerrero-Fajardo, Mayra Estacio, Alexis A. Franco

**Affiliations:** 1 Departamento Materno-infantil, Unidad de Trasplante de Médula Ósea, Fundación Valle del Lili, Cali, Colombia Fundación Valle del Lili Cali Colombia; 2 Facultad de Ciencias de la Salud, Universidad Icesi, Cali, Colombia Universidad Icesi Universidad Icesi Cali Colombia; 3 Departamento de Psiquiatría, Fundación Valle del Lili, Cali, Colombia Fundación Valle del Lili Cali Colombia; 4 Centro de Investigaciones Clínicas, Fundación Valle del Lili, Cali, Colombia Fundación Valle del Lili Cali Colombia

**Keywords:** trasplante de médula ósea, calidad de vida, niño, salud mental, Bone marrow transplantation, quality of life, child, mental health

## Abstract

**Introducción.:**

El trasplante de células madre hematopoyéticas es la opción curativa para algunas enfermedades y está aumentando el tiempo de supervivencia de los pacientes. La calidad de vida relacionada con la salud en estos pacientes no se evalúa de manera sistemática.

**Objetivos.:**

Describir la calidad de vida relacionada con la salud y las complicaciones en niños con trasplante de células madre hematopoyéticas.

**Materiales y métodos.:**

Es un estudio transversal en pacientes pediátricos sobrevivientes al trasplante. Se midió la calidad de vida relacionada con la salud, utilizando el cuestionario KIDSCREEN-27 en pacientes entre 8 y 14 años y la SF-12™ (*Short Form-12*) en pacientes mayores de 14 años. El análisis estadístico se realizó en el *software* Stata 12. Utilizamos el modelo de Rasch, trasladando estimación de parámetros a valores t para obtener el resultado de los cuestionarios.

**Resultados.:**

En total, 42 pacientes respondieron alguno de los cuestionarios. Los eventos adversos más frecuentes fueron “enfermedad crónica de injerto *Vs*. Contra huésped” y “complicaciones endocrinas”. De acuerdo con la normalidad de datos del KIDSCREEN-27, los puntajes de las dimensiones “ambiente escolar” y “soporte social y pares” fueron inferiores al percentil 50. En el cuestionario SF-12™, el grupo que utilizaba inmunosupresores tuvo un menor puntaje en el componente físico.

**Conclusiones.:**

En general, los resultados del KIDSCREEN-27 sugieren un cierto déficit de calidad de vida en pacientes entre 8 y 14 años. Los cuestionarios mostraron confiabilidad en la muestra.

## Abstract (en)

**Introduction:**

Hematopoietic stem cell transplantation is the curative option for some diseases and is increasing patient survival. The health-related quality of life in these patients is not systematically evaluated.

**Objectives:**

The present study sought to describe the health-related quality of life and complications in children who underwent hematopoietic stem cell transplantation.

**Materials and methods:**

A cross-sectional study was conducted on pediatric transplanted survivors. Health-related quality of life was measured using the KIDSCREEN-27 scale and Short Form-12 (SF-12) in patients between 8 and 14 years of age and those over 14 years, respectively. Statistical analysis was performed using STATA 12 software. We used the Rasch model person parameter estimates translated into T-values to score the questionnaire.

**Results:**

A total of 42 children answered the questionnaires. The most frequent adverse events were chronic graft *Vs*. host disease and endocrine complications. According to European norm data in the KIDSCREEN-12 scale, scores for the school dimension and social and peer support were below the 50th. percentile. The group administered immunosuppressants had lower scores on the physical component of the SF-12™ scale.

**Conclusions:**

In general, the KIDSCREEN-27 does appear to suggest some quality-of-life deficit in younger children. The scales showed reliability in this population.

El trasplante de progenitores hematopoyéticos se considera la opción curativa en varias enfermedades, incluyendo benignas y malignas. A lo largo del tiempo, las modificaciones en la técnica han logrado disminuir las complicaciones posteriores al trasplante y la mortalidad. El incremento de la supervivencia ha expuesto a los pacientes a complicaciones a largo plazo ^1^, como enfermedad de injerto contra huésped, uso prolongado de inmunosupresores, complicaciones endocrinas, compromiso cardiopulmonar o alteraciones musculoesqueléticas ^2^, lo cual repercute en el crecimiento y el desarrollo de los pacientes ^3^. La ocurrencia de, al menos, una condición crónica se estima en dos terceras partes de los pacientes con trasplante, y se ha reportado afectación grave en una quinta parte de ellos ^2^.

La expresión “calidad de vida relacionada con la salud” ^4^ fue propuesta para nombrar el grado de bienestar derivado de la evaluación de diferentes dominios de la vida que reflejan el estado de salud ^5^^,^^6^. La validación y adaptación de cuestionarios son típicamente usados para evaluar la calidad de vida relacionada con la salud ^3^^,^^7^. Entre ellos está KIDSCREEN-27, un cuestionario alemán validado para pacientes pediátricos colombianos y utilizado para evaluar niños y adolescentes sanos o con enfermedades crónicas, incluyendo aquellos con cáncer ^8^. Otro cuestionario ampliamente utilizado es el SF-12, que corresponde a la versión corta del SF-36 y está validado para pacientes crónicos mayores de 14 años ^9^. Aunque la validación de cuestionarios no siempre es posible, existen herramientas para determinar su confiabilidad, como el alfa de Cronbach ^10^.

A pesar de la disponibilidad de cuestionarios, pocos estudios se enfocan en los pacientes pediátricos con trasplantes. Desde nuestro conocimiento, no existen estudios que evalúen la calidad de vida en niños con trasplante en Colombia. En este estudio, se quiere describir la calidad de vida relacionada con la salud después del trasplante de células madre hematopoyéticas en pacientes pediátricos y las complicaciones a largo plazo.

## Materiales y métodos

Se realizó un estudio transversal en la Fundación Valle del Lili, un centro de referencia para trasplantes de médula ósea, en Cali (Colombia), en pacientes menores de 18 años que recibieron el trasplante entre enero de 2012 y diciembre de 2017. Los supervivientes fueron contactados por vía telefónica o en una entrevista personal en mayo de 2018, para responder el cuestionario. Los pacientes fallecidos o los que no respondieron el cuestionario, fueron excluidos.

Este estudio fue aprobado por el Comité de Ética en Investigación Biomédica de la Fundación Valle del Lili, el 9 de mayo de 2018 con el número 1239. Se diligenciaron los consentimientos informados de acuerdo con la Declaración de Helsinki.

Los datos se obtuvieron de los registros de historia clínica. La información incluyó complicaciones posteriores al trasplante de los sistemas cardiopulmonar, gastrointestinal, renal, nervioso central o musculoesquelético, enfermedad crónica de injerto contra huésped y neoplasias malignas subsecuentes.

La calidad de vida fue medida usando el cuestionario KIDSCREEN-27 en pacientes entre 8 y 14 años, y el SF-12v2™ para los mayores de 14 años. Los cuestionarios se administraron independientemente del transcurrido desde el momento del trasplante.

El KIDSCREEN-27 consta de 27 preguntas divididas en cinco dominios: bienestar físico, bienestar psicológico, relaciones con los padres y autonomía, soporte social y pares, y ambiente escolar ^11^. Los elementos se puntúan en una escala de Likert de 5 puntos con tres conjuntos de respuestas diferentes: i) poco, un poco, bueno, muy bueno, excelente; ii) nada, un poco, moderadamente, mucho, muchísimo; iii) nunca, casi nunca, algunas veces, casi siempre, siempre.

El SF-12v2™ es un cuestionario para evaluar la calidad de vida relacionada con la salud, basado en 12 preguntas para medir 8 dominios en salud, y obtener información sobre el componente resumen de salud física y mental. Los dominios relacionados con la salud física incluyen salud general, funcionamiento físico, rol físico y dolor corporal. Los que corresponden a la salud mental son vitalidad, funcionamiento social, rol emocional y salud mental ^12^. Ambos cuestionarios han sido validados para enfermedades crónicas, incluyendo el cáncer pediátrico ^9^^,^^11^.

### Análisis estadístico

Se hizo un análisis descriptivo en todas las variables utilizadas. Para las variables categóricas, se utilizaron frecuencias absolutas y relativas. En las variables continuas, se usaron promedios y desviación estándar (DE), medianas y rangos intercuartílicos (RIC). El *software* estadístico para el análisis fue STATA 12 (StataCorp, Texas, USA).

Se desarrolló un subanálisis para los pacientes con enfermedad crónica de injerto contra huésped, los que recibieron inmunosupresores y aquellos con enfermedad neoplásica o no neoplásica. Se consideró significativo un valor de p<0,05. Se calculó el alfa de Cronbach para evaluar la fiabilidad de los cuestionarios en esta población, basándose en las dimensiones descritas en la validación original del cuestionario KIDSCREEN-27 ^8^ y los componentes del SF-12.

El KIDSCREEN-27 fue analizado usando el paquete estadístico SPSS™, puntuando el cuestionario por el modelo de Rasch y trasladándolos a valores T con un promedio de 50 y una DE de 10. Los puntajes más altos reflejan una mejor calidad de vida.

El SF-12v2™ fue analizado con un algoritmo específico, decodificando cada respuesta por un dominio y obteniendo las medidas de los componentes resumen (físico y mental). La interpretación se simplificó con el puntaje de normalidad de datos con un promedio de 50 y DE de 10. Los valores más altos o bajos corresponden a mejores o peores resultados, de acuerdo con la población de referencia.

## Resultados

**Figura 1:**
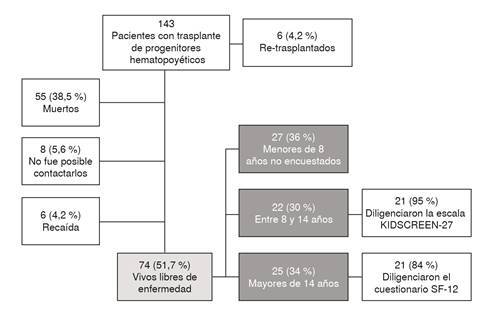
Diagrama de flujo de la selección de pacientes para diligenciar la encuesta

Entre los años 2012 y 2017, se realizaron 149 trasplantes de médula ósea en 143 pacientes, en 6 de ellos en forma reiterada. Al momento de contactar a los pacientes (mayo de 2018), había 55 fallecidos (38 %), 8 (6 %) no tenían información disponible y 6 estaban en recaída (4 %). Los pacientes vivos y libres de enfermedad eran 74 (52 %); de estos, 27 (36,5 %) eran menores de 8 años y 47 (63,5 %) eran mayores de 8 años. Las encuestas se llevaron a cabo en 42 (56,7 %) pacientes mayores de 8 años. La selección de los pacientes se muestra en un diagrama de flujo (figura 1).

La mediana del tiempo de seguimiento fue de 31 meses (RIC=15-54). La edad promedio al momento del seguimiento fue de 11 años (DE=5). Hubo 27 (57,4 %) pacientes de sexo masculino.

Los diagnósticos encontrados para indicar el trasplante fueron: leucemia linfoblástica aguda en 22 (46,8 %) pacientes, anemia de células falciformes en 7 (14,9 %), leucemia mieloide aguda en 6 (12,8 %), anemia aplásica en 5 (10,6 %), tumores sólidos en 3 (6,4 %), inmunodeficiencia primaria en uno (2,1 %), síndrome mielodisplásico en uno (2,1 %), leucemia mieloide crónica en uno (2,1 %) y linfoma no Hodgkin en otro (2,1 %). En 43 (91,4 %) pacientes se practicó un trasplante alogénico y, en 4 (8,5 %), uno autólogo. Las características de los pacientes y del trasplante, se muestran en el cuadro 1.

**Cuadro 1:** Características sociodemográficas en 47 pacientes con trasplante

Característica			
Edad al trasplante en años			
	Media (DE)	11 (5)
	Rango	1 a 23
Sexo masculino, n (%)		27 (57,4)
Indicación de trasplante		n (%)
	Leucemia linfoblástica aguda	22 (46,8)
	Leucemia mieloide aguda	6 (12,8)
	Anemia de células falciformes	7 (14,9)
	Inmunodeficiencia primaria	1 (2,1)
	Tumores sólidos	3 (6,4)
	Anemia aplásica	5 (10,6)
	Síndrome mielodisplásico	1 (2,1)
	Leucemia mieloide crónica	1 (2,1)
	Linfoma no Hodgkin	1 (2,1)
Tipo de trasplante		
	Haploidéntico	30 (63,8)
	Idéntico	12 (25,5)
	Autólogo	4 (8,5)
	Cordón umbilical	1 (2,1)
Tiempo de seguimiento (meses)		
	Mediana (RIC)	31 (15-54)
	Rango	5 a 78

DE: desviación estándar; RIC: rango intercuartílico

Las complicaciones a largo plazo posteriores al trasplante al momento del seguimiento se dividieron en categorías. Hubo 2 (4,2 %) pacientes con enfermedad cardiaca, 19 (40,4 %) con enfermedad crónica de injerto contra huésped, 8 (17 %) con síntomas pulmonares crónicos, 12 (25,5 %) con complicaciones del sistema endocrino, 3 (6,4 %) con dolor musculoesquelético, 2 (4,2 %) con compromiso renal, 1 (2,1 %) con compromiso auditivo, 5 (10,6 %) con compromiso hepático, y 1 (2,1 %) con compromiso neurológico; además, 25 (53,2 %) recibieron medicamentos adicionales para el manejo de las comorbilidades (cuadro 2).

**Cuadro 2:** Complicaciones en 47 pacientes trasplantados

Característica		n (%)
Enfermedad cardiaca		2 (4,2)
	Hipertensión arterial sistémica	1 (50)
	Enfermedad valvular	1 (50)
Enfermedad de injerto contra huésped		19 (40,4)
	Piel	8 (42,1)
	Ojos	1 (5,3)
	Aparato gastrointestinal	1 (5,3)
	Hígado	3 (15,7)
	Musculoesquelético	1 (5,3)
	Pulmón	4 (21)
	Sistema hematopoyético	1 (5,3)
Enfermedad de injerto contra huésped en el último año		17 (36,2)
Síntomas crónicos pulmonares		8 (17)
Complicaciones del sistema endocrino		12 (25,5)
	Talla baja	4 (33,3)
	Disfunción tiroidea	1 (8,3)
	Osteoporosis	2 (16,7)
	Amenorrea	5 (41,7)
Complicaciones por dolores musculoesqueléticos		3 (6,4)
Compromiso renal		2 (4,2)
Pérdida de la audición		1 (2,1)
Compromiso hepático		5 (10,6)
Síndrome convulsivo		1 (2,1)
Uso de medicamentos		25 (53,2)
	Antidepresivos	0
	Anticonvulsivos	1 (4)
	Analgésicos	1 (4)
	Hormonas	7 (28)
	Inmunosupresores	12 (48)
	Antihipertensivos	1 (4)
	Inhaladores	3 (12)

El cuestionario SF-12 fue diligenciado por 21 pacientes. Los dominios físico y mental tuvieron un promedio de 52,2 (DE=7,3) y de 52,3 (DE=10,3), respectivamente. Hubo 5 pacientes con enfermedad crónica de injerto contra huésped que llenaron el cuestionario SF-12 y obtuvieron un promedio de 52 (DE=8,4) en el componente físico y uno de 52,6 (DE=10,8) en el componente mental. El uso de inmunosupresores se registró en 4 pacientes con el cuestionario SF-12 y los promedios fueron de 46,4 (DE=6,8) para el componente físico y de 53,1 (DE=6,2) para el mental. En los pacientes (n=16) con enfermedad neoplásica, el promedio en el componente físico fue de 53 (DE=7) y, en el mental, de 51,7 (DE=10,9). Ninguna de las relaciones fue estadísticamente significativa. En el cuadro 3 se muestran los resultados de cada cuestionario.

**Cuadro 3:** Cuestionarios sobre calidad de vida relacionada con la salud, en pacientes con trasplante y enfermedad de injerto contra huésped crónica, uso de inmunosupresores o enfermedad neoplásica

Característica SF-12		Total (n=21)	cEICH (n=5)	p	Uso de inmunosupresores (n=4)	p	Enfermedad neoplásica (n=16)	^p^
Resumen componente físico								
Media (DE)		52,2 (7,3)	48,2 (7,1)		46,4 (6,8)		53 (7)	
Mediana (RIC)		53,3 (48,6-57,6)	51 (42,2-53,3)	0,17	46,6 (40,6-52,1)	0,08	53,4 (49-57,6)	0,3
Resumen componente mental								
Media (DE)		52,3 (10,3)	52,6 (10,8)	0,9	53,1 (6,2)	0,8	51,7 (10,9)	0,7
Mediana (RIC)		52,1 (46,1-62,4)	52,9 (51-55)		52,9 (48,6-57,6)		53,4 (46,9-61,5)	
Característica		Total, n=21	cEICH, n=13	P	Uso de inmunosupresores, n=8	p	Patología neoplásica, n=14	p
	KIDSCREEN-27							
Bienestar físico								
	Tiempo							
	Media (DE)	51,8 (14,3)	52,1 (15,5)		53,6 (14,7)		55,7 (13,2)	
	Mediana (RIC)	52,4 (49,6-64,3)	52,4 (49,6-64,3) 0,7		52,4 (44,8-64,3)	0,8	54 (49,6-64,3)	0,1
Bienestar psicológico								
	Tiempo							
	Media (DE)	62 (41,2)	51,1 (12,9)		49,3 (13,3)		54,9 (12,3)	
	Mediana (RIC)	48,4 (44,8-64,3)	48,4 (43,2-64,3)		45 (42,8-56,4)	0,3	54 (46,5-64,3)	0,2
Relación con los padres y autonomía								
	Tiempo							
	Media (DE)	66,1 (52,7)	54,9 (12,8)		50,9 (5,8)		56,5 (13,3)	
	Mediana (RIC)	51,2 (48-64)	51,2 (46,5-59)	0,2	50,3 (45,9-56,1)	0,3	52,2 (49,5-74,4)	0,2
Soporte social y pares								
	Tiempo							
	Media (DE)	48,5 (13,7)	46,4 (14,6)		41,2 (16)		50,8 (10,3)	
	Mediana (RIC)	53,2 (44,4-57,8)	53,2 (37,8-53,2)	0,2	42,4 (32,5-55,5)	0,3	53,2 (44,4-57,8)	0,4
Ambiente escolar								
	Tiempo							
	Media (DE)	40,4 (21,8)	34,5 (23,9)	0,1	30,1 (22)		44,4 (22)	
	Mediana (RIC	45,4 (16,3-58,1)	16,3 (16,3-58,1)	1	16,3 (16,3-44,3)	0,09	52,7 (16,3-58,2)	0,2

DE: desviación estándar; RIC: rango intercuartílico; cEICH: enfermedad crónica de injerto contra huésped

Las medias se compararon con la prueba t de Student.

Los resultados promedio por dimensión fueron los siguientes: bienestar físico, 51,8 (DE=14,3), bienestar psicológico, 62 (DE=41,2), relaciones con los padres y autonomía, 66,1 (DE=52,7), soporte social y pares, 48,52 (DE=13,66) y, ambiente escolar, 40,4 (DE=21,8). Al comparar con la normalidad de datos europeos, la dimensión de soporte social y pares se encontró entre los percentiles 25 a 50 y, la de ambiente escolar, entre los percentiles 10 a 25 ^11^. De estos 21 pacientes, 13 (62 %) tenían enfermedad crónica de injerto contra huésped, 8 (38 %) usaban inmunosupresores y 14 (67 %) tenían patología neoplásica, en los tres subanálisis se obtuvieron puntajes más bajos en las dimensiones de soporte social y pares, y ambiente escolar. La comparación de los promedios para soporte social y pares, entre el uso (41 ± 5,6) o no uso de inmunosupresores (54 ± 2,6), mostró una diferencia estadísticamente significativa (p=0,03), lo cual indica una menor calidad de vida para esta dimensión en aquellos pacientes con inmunosupresión. El alfa de Cronbach mostró que los cuestionarios fueron confiables para esta población (cuadro 4).

**Cuadro 4:** Análisis de Cronbach’s Alpha para los cuestionarios KIDSCREEN-27 Y SF-27

	KIDSCREEN-27	Dimensión 1	Dimensión 2	Dimensión 3	Dimensión 4	Dimensión 5	**SF-12**
Alfa de Cronbach	0,9197	0,9098	0,8565	0,8264	0,8890	0,9496	0,8901
Estandarizado	0,9303	0,9096				0,9499	0,8925

## Discusión

En los últimos años, las mejoras en la técnica del trasplante de células madre hematopoyéticas para el tratamiento de enfermedades benignas y malignas en población pediátrica, han aumentado el número de supervivientes. Las complicaciones a largo plazo y los efectos multidimensionales en la calidad de vida relacionada con la salud después del procedimiento han sido focos de interés para investigar.

En el presente estudio, se describen las complicaciones a largo plazo después del trasplante en 47 pacientes; y se utilizaron los cuestionarios KIDSCREEN-27 y SF-12™ en 42 pacientes para evaluar la calidad de vida relacionada con la salud. Estos resultados revelan que los niños entre 8 y 14 años reportaron menores puntajes en los dominios de ambiente escolar, y soporte social y pares, en contraste con los mayores de 14 años, quienes obtuvieron puntajes por encima del promedio de la normalidad de base. Sin embargo, los niños con enfermedad crónica de injerto contra huésped y uso de inmunosupresores, reportaron menores puntajes en la dimensión de ambiente escolar. Ambos cuestionarios mostraron fiabilidad en esta población.

Los pacientes con trasplante experimentan un considerable deterioro en su estado de salud, no sólo por la condición clínica, sino también, en términos de calidad de vida, funcionalidad y estado de ánimo, ya que experimentan una mayor carga emocional y aumentan la ansiedad y el estrés ^13^. Por ello, en los últimos años, los profesionales de la salud han tomado conciencia de la importancia de brindar una mejor calidad de vida y han desarrollado equipos interdisciplinarios para evaluar estos factores ^14^^,^^15^.

Durante el seguimiento clínico de estos pacientes, es indispensable evaluar la presencia de complicaciones a largo plazo, debido a que estas pueden empeorar el estado de salud y la calidad de vida. Una de las mayores preocupaciones en los pacientes con trasplante es la enfermedad crónica de injerto contra huésped y el uso prolongado de inmunosupresores, lo cual se encuentra con frecuencia en la práctica clínica y requiere mayor atención médica.

Liu, *et al*. ^16^, encontraron que existe una correlación negativa entre la presencia de esta enfermedad crónica y la calidad de vida al afectar el funcionamiento emocional y social, evaluado por medio del *Paediatric Quality of Life Inventory*™ (PedsQL 4.0™), cuyas dimensiones son similares a las del KIDSCREEN-27 ^17^. Sin embargo, Jensen, *et al*. ^18^, no encontraron asociaciones significativas con el cuestionario SF-36, posiblemente debido a la poca frecuencia de la enfermedad de injerto contra huésped en ese estudio. Por otra parte, Kurosawa, *et al*. ^19^, mostraron que el tratamiento inmunosupresor afecta la calidad de vida, en especial, el componente social.

En el presente estudio, la enfermedad crónica de injerto contra huésped en menores de 14 años y los inmunosupresores en los mayores de 14 años disminuyeron los puntajes de ambiente escolar y el componente físico, respectivamente. Además, Schultz, *et al*. ^20^, mencionaron la importancia de tener en cuenta las condiciones crónicas de los sobrevivientes después de un trasplante de médula ósea, y mostraron que las comorbilidades cardiovasculares y endocrinas son las más comunes. Estos hallazgos son parcialmente similares a los nuestros, en los cuales las complicaciones endocrinas estuvieron entre las más frecuentes.

En el KIDSCREEN-27, se encontró que en comparación con los datos de normalidad europea ^11^, los pacientes entre los 8 y los 14 años obtuvieron un menor puntaje en la dimensión de ambiente escolar (percentiles de 10 a 25), y en la de soporte social y pares (percentiles de 25 a 50). Las demás dimensiones se mantenían en un puntaje promedio, igual o mejor que los percentiles de 50 a 75. En otro estudio, también se mencionó afectación de las dimensiones del entorno escolar, y de soporte social y pares, considerándose como posible explicación que los niños que repiten años escolares deben empezar a socializar de nuevo al reintegrarse ^21^.

En el presente estudio, se ven positivamente los hallazgos de las dimensiones con puntajes iguales o mejores que la normalidad y se quiere abrir la posibilidad de ideas para mejorar las dimensiones con menores puntajes. Una de las soluciones implementadas en Taiwán para garantizar la escolarización de los pacientes que no pueden asistir a clases presenciales debido a tratamientos o enfermedades, es brindar un programa de educación especial en estos casos ^16^.

Las publicaciones en Latinoamérica sobre calidad de vida relacionada con la salud en pacientes pediátricos son escasas. Encontramos un estudio brasilero en el que se evaluó la condición de funcionalidad después del trasplante en adolescentes mediante la escala de Lansky o de Karnosfky ^22^. En México, usaron el *Pediatric Cancer Quality of Life Inventory-3* (PCQL-3) en niños con leucemia linfoblástica aguda durante el tratamiento ^23^ y, en otro estudio en Chile, se utilizó el cuestionario KIDSCREEN-27 en niños que recibieron tratamiento curativo para leucemia linfoblástica aguda con el protocolo PINDA ^5^.

En Colombia, se han realizado validaciones y revisiones de escalas en los últimos años ^4^, pero no se encontraron estudios sobre la calidad de vida después del trasplante de médula ósea en población pediátrica. Aunque en el presente estudio no fue posible hacer una validación, el cuestionario KIDSCREEN-27 ya ha sido validado en Colombia en niños y adolescentes sanos o con enfermedades crónicas ^8^; esto sugiere que el cuestionario es culturalmente aplicable en niños colombianos ^24^.

En el cuestionario SF-12™, se encontró que los dos componentes resumen mostraron un estado de salud igual o mejor que el promedio; sin embargo, el componente físico disminuyó ligeramente en pacientes con inmunosupresión. En otros estudios en que se evaluó la calidad de vida mediante cuestionarios similares, se concluyó que, a mayor tiempo de supervivencia después de un trasplante alogénico en la infancia, los sobrevivientes alcanzan resultados similares a los de la población normal ^18^^,^^25^^,^^26^. Sin embargo, en otro se demostró que los adultos que sobrevivieron a una enfermedad maligna en la infancia obtuvieron menores puntajes en el componente psicológico ^1^. Para aclarar estos hallazgos, en futuras investigaciones se debería tener en cuenta el tiempo entre el trasplante y la encuesta, y el tiempo de supervivencia.

Este estudio tiene limitaciones. En este diseño, los cuestionarios se usaron en un solo centro y en un solo momento, lo cual permitió la variabilidad de los periodos posteriores al trasplante de médula ósea. Aunque no hay validaciones de los cuestionarios en pacientes con trasplante, sí las hay en pacientes pediátricos con enfermedades crónicas, y el cálculo del coeficiente alfa de Cronbach reveló la coherencia interna de los cuestionarios.

El trasplante alogénico de células madre hematopoyéticas puede causar complicaciones a largo plazo que afecten la calidad de vida. Los resultados del KIDSCREEN-27 sugieren un cierto déficit de calidad de vida en pacientes entre 8 y 14 años. Sin embargo, es necesario continuar investigando con diseños prospectivos para conocer los efectos a largo plazo de este tipo de trasplante en relación con la calidad de vida y ajustar la terapia de soporte de los pacientes.
